# Neurological recovery after liver transplantation in a severely disabled adolescent with Wilson disease: a 1-year follow-up case report

**DOI:** 10.3389/fped.2026.1862229

**Published:** 2026-06-17

**Authors:** Konstantin Semash, Timur Dzhanbekov, Mansur Nasirov, Alla Syrkina, Bakhtiyarjon Umarov

**Affiliations:** 1 Department of Minimally Invasive Surgery and Transplantation, National Children’s Medical Center, Tashkent, Uzbekistan; 2 Department of Liver Transplantation, V. I. Shumakov Transplantology & Artificial Organs National Medical Research Center, Moscow, Russia

**Keywords:** case report, liver transplantation, neurological recovery, neuro-Wilson disease, UWDRS, Wilson disease

## Abstract

**Background:**

Neurological Wilson disease with severe motor, cognitive, and functional impairment remains a challenging and controversial indication for liver transplantation because neurological injury is often considered irreversible. The Unified Wilson Disease Rating Scale (UWDRS) is used to quantify disease severity and provides an objective framework for monitoring neurological outcomes.

**Case presentation:**

A 15-year-old adolescent with a 5-year history of Wilson disease presented with rapid neurological deterioration, profound malnutrition, tense ascites, hepatic decompensation, and severe neurological dysfunction including immobility, loss of speech, contractures, sialorrhea, dysphagia, and encephalopathy. The total UWDRS score at presentation was 162. Liver transplantation was performed as a life-saving procedure. The postoperative course was complicated by aspiration pneumonia requiring reintubation and prolonged enteral feeding. Continuous neuropsychosocial rehabilitation was implemented in the intensive care unit, the transplant ward, and during outpatient follow-up.

**Outcome:**

At 1 year, the patient demonstrated marked recovery. UWDRS scores improved to 59, speech returned, cognitive engagement normalized, contractures decreased, and she regained the ability to walk with support and perform basic daily activities with assistance and returned to school.

**Conclusion:**

This case provides evidence that significant neurological improvement is possible in adolescents with advanced neuro-Wilson disease following timely liver transplantation. Restoration of copper metabolism combined with structured rehabilitation may lead to substantial functional recovery even in patients with severe preoperative neurological compromise. This case supports the view that severe neurological impairment should not be considered an absolute contraindication to liver transplantation in carefully selected patients.

## Introduction

Wilson disease is an inherited disorder of copper metabolism that may present with hepatic, neurological, or psychiatric manifestations (1). The neurological form is particularly challenging, as progressive involvement of the basal ganglia can lead to severe and potentially irreversible motor, cognitive, and behavioral impairment (2). The severity of neurological dysfunction is commonly quantified using the Unified Wilson Disease Rating Scale (UWDRS), which provides structured assessment across neurological, hepatic, and psychiatric domains and is increasingly used to evaluate disease burden and outcomes (3).

In patients with advanced neuro-Wilson disease, the role of liver transplantation remains controversial. Many centers consider the procedure high-risk or even unsuitable when profound neurological deficits are present, largely due to concerns about irreversible central nervous system injury and uncertain potential for recovery (4). Reports of meaningful neurological improvement after transplantation are relatively rare, and each documented case contributes valuable insight into the reversibility of neurological damage once copper metabolism is corrected (5).

We present the case of a severely debilitated adolescent with advanced neurological Wilson disease, extremely high UWDRS scores, and life-threatening hepatic decompensation, who experienced substantial neurological and functional recovery following living donor liver transplantation. This case highlights the importance of individualized decision-making and adds to the growing evidence that even marked neurological impairment may not represent an absolute contraindication to transplantation.

## Case presentation

The present case report adheres to the CARE guidelines for clinical case reporting. Written informed consent for publication was obtained from the patient, who reviewed the manuscript and approved the use of anonymized clinical data and imaging.

A 15-year-old female with a 5-year history of Wilson disease, initially diagnosed after the appearance of early neurological symptoms, was admitted to our center in a state of profound clinical deterioration. The diagnosis of Wilson disease in this patient had been previously confirmed by comprehensive biochemical and genetic evaluation. Genetic testing revealed two pathogenic mutations in the *ATP7B* gene, establishing the diagnosis at the molecular level. Biochemical studies repeatedly demonstrated reduced ceruloplasmin levels, elevated serum copper, and persistently increased urinary copper excretion. Slit-lamp examination consistently showed the presence of Kayser–Fleischer rings. According to the Leipzig scoring system, the patient scored 10 points, meeting diagnostic criteria for Wilson disease with high certainty. The patient had been treated with D-penicillamine for several years; however, adherence was poor, with frequent missed doses. Family history was remarkable for the death of her biological sister from cirrhosis secondary to untreated Wilson disease.

Upon presentation, the patient demonstrated massive upper gastrointestinal bleeding, severe cachexia with a BMI of 14.9, ascites, respiratory distress, oliguria, and clear signs of advanced liver failure. Her height was 186 cm and weight 51 kg. Laboratory tests confirmed hepatic decompensation with ALT 89 U/L, AST 110 U/L, total bilirubin 50 µmol/L, direct bilirubin 28 µmol/L, INR 2.5, albumin 28 g/L, creatinine 110 µmol/L, urea 15.1 mmol/L, ammonia 17 µmol/L, and ceruloplasmin 25 mg/dL. MELD-Na was calculated at 23, and she met criteria for Child–Pugh Class C. Abdominal ultrasound and CT revealed a markedly reduced and nodular liver consistent with cirrhosis and large-volume tense ascites. Portal hypertension was severe, with grade III esophageal varices identified on endoscopy. Brain MRI demonstrated T2/FLAIR hyperintensity of the caudate nuclei, a finding characteristic of copper accumulation in neurological Wilson disease (Figure 1). Neurologically, the patient presented with pronounced encephalopathy, peripheral polyneuropathy, severe gait and speech disturbances, sialorrhea, and fixed contractures of all extremities. Emergency endoscopic hemostasis of variceal bleeding was performed in the intensive care unit. Refractory ascites persisted despite medical therapy, requiring repeated paracentesis with a cumulative evacuation of approximately 30 L. Nutritional rehabilitation included albumin and plasma transfusions, high-calorie enteral supplementation for cachexia, and subsequent nasogastric tube feeding due to the decline of neurological function. After multidisciplinary discussion and detailed counseling of the family, a decision was made to proceed with living donor transplantation.

**Figure 1:**
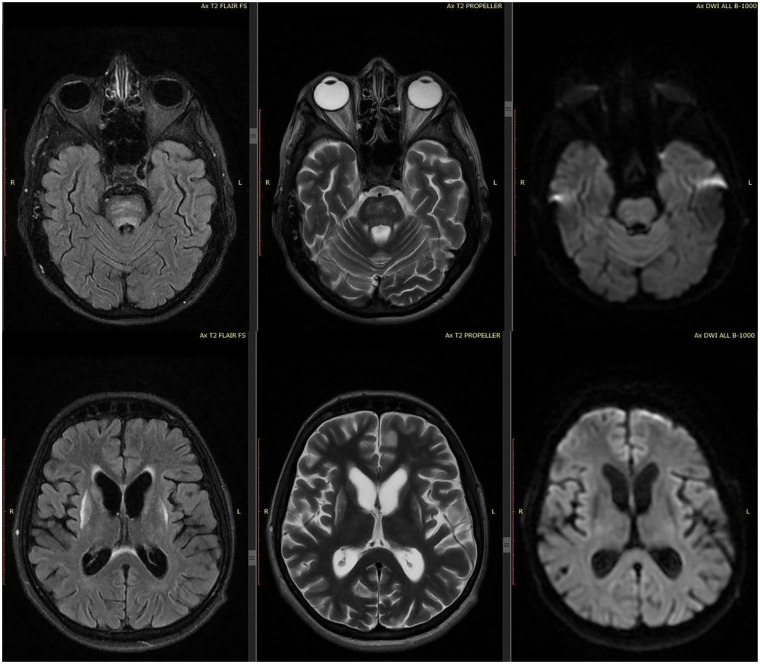
Brain MRI of the patient on admission. T2/FLAIR hyperintensity of the caudate nuclei, a finding characteristic of copper accumulation in neurological Wilson disease.

A living-related right lobe liver transplantation was performed with the patient's uncle serving as the donor. The graft-to-recipient weight ratio was 1.5%. The operation lasted 700 min, with cold ischemia time of 1 h 50 min and warm ischemia time of 30 min. Immunosuppressive therapy followed our standard institutional protocol (6).

The initial postoperative course was stable. On the fourth postoperative day, however, the patient developed acute respiratory deterioration. Imaging and clinical findings confirmed aspiration pneumonia associated with her pre-existing impaired airway protection. She required re-intubation and mechanical ventilation until postoperative day 10. Antimicrobial therapy included meropenem and linezolid, with inhaled colistin administered as adjunctive treatment. No bronchoscopy was performed; airway clearance was achieved with repeated suctioning. Non-invasive ventilation after extubation was not required. On postoperative day 13, she was transferred to the transplant ward. Enteral tube feeding was continued until safe swallowing was restored. Graft function remained satisfactory, surgical complications did not occur, and the patient was discharged on postoperative day 22.

Throughout the entire hospitalization, the patient underwent comprehensive neuro–psycho–social rehabilitation. In the intensive care unit, she received inhalational therapy consisting of nebulized bronchodilators (Berodual®), inhaled budesonide (Pulmicort®), and inhaled colistin as adjunctive antimicrobial treatment, in addition to postural drainage, respiratory physiotherapy, therapeutic massage, and passive mobilization. After transfer to the ward, the rehabilitation program expanded to include physiotherapy with vibratory massage, structured breathing exercises, continued inhalational therapy, and progressive physical therapy adapted to her neurological condition.

Approximately 2 months after discharge, the patient experienced a secondary neurological deterioration. Muscle weakness progressed, a wing-beating tremor emerged, speech became increasingly dysarthric, and new swallowing difficulties developed, necessitating the reintroduction of nasogastric tube feeding. She was readmitted for further evaluation. At the time of neurological deterioration, no evidence of graft dysfunction was identified. Liver biochemistry remained stable, with ALT 63 U/L, AST 50 U/L, total bilirubin 9.6 μmol/L, and INR 1.1. Tacrolimus trough concentration was 7.0 ng/mL and remained within the therapeutic range. Follow-up brain MRI demonstrated interval worsening, with new hyperintense T2/FLAIR signal abnormalities in the bilateral putamen, globus pallidus, and pontine region, findings consistent with acute copper redistribution or delayed neurotoxicity (Figure 2). D-penicillamine therapy was reinitiated, resulting in gradual clinical improvement and stabilization of neurological symptoms. Following discharge, she was monitored by a neurologist and continued systematic therapeutic exercise aimed at restoring mobility, coordination, and cognitive function, with gradual reintegration into everyday activities and social interactions. D-penicillamine was discontinued after 6 months because of stable graft function, sustained neurological improvement, and absence of further neurological progression. Clinical evaluation after transplantation demonstrated substantial improvement. Preoperative UWDRS scores were 162. One-year follow-up scores had improved to 59 reflecting marked neurological recovery. Follow-up brain MRI demonstrated radiological improvement. There was a clear reduction of the previously observed T2/FLAIR hyperintensity within the pons, indicating partial resolution of the acute changes noted earlier. Hyperintense signal abnormalities in the claustrum and putamen persisted, more pronounced on the right side; however, their extent and intensity had decreased compared with prior imaging, consistent with gradual regression of copper-related neurotoxicity (Figure 3).

**Figure 2:**
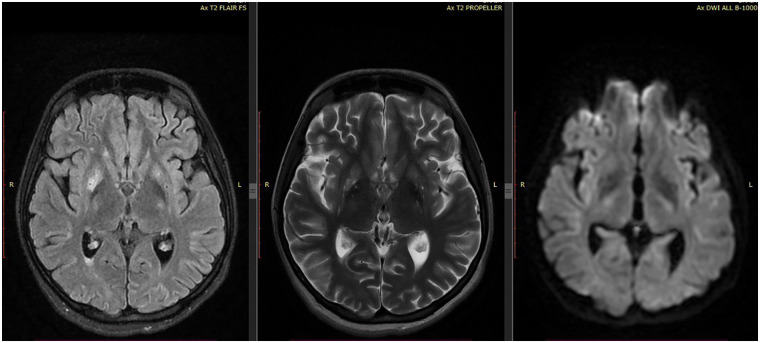
Marked bilateral T2/FLAIR hyperintensity of the claustrum, putamen, and pontine region, findings that may indicate acute copper redistribution or neurotoxicity.

**Figure 3:**
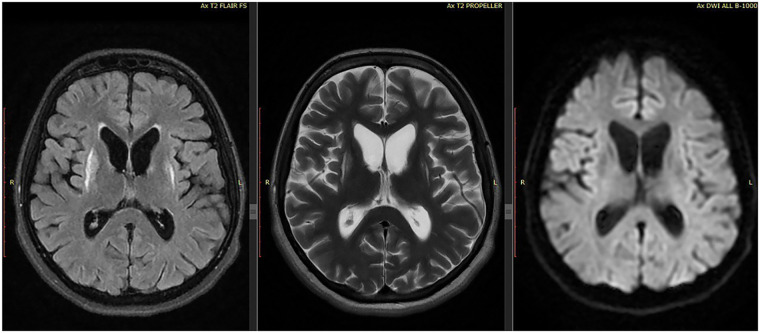
MRI shows a clear reduction of the previous T2/FLAIR hyperintensity in the pons. Hyperintense signal in the claustrum and putamen persists, more on the right side, but both the extent and intensity have decreased compared with earlier imaging, which is consistent with gradual regression of copper-related neurotoxicity.

Functionally, the patient regained the ability to walk with support, developed fluent speech with minimal residual defects, became fully oriented, and showed a notable reduction in sialorrhea and contractures. She is able to eat independently, perform basic activities of daily living with minor assistance, returned to school, and actively engage in social interactions with her family and peers.

The chronological evolution of clinical status, neurological manifestations, liver graft function, major therapeutic interventions, and outcomes throughout follow-up is summarized in Table 1. The patient's family shared that they had perceived her rapid neurological decline, including the loss of speech, mobility, and independence, as irreversible. They describe her postoperative recovery as a profound return to communication and daily activities and consider the transplant to have given her a renewed chance at meaningful life.

**Table 1:** Clinical timeline and neurological recovery after liver transplantation.

Time point	Clinical status	Neurological findings	Liver/Graft status	Key interventions
Five years before LT	Diagnosis of Wilson disease	Initial neurological manifestations	Preserved liver function	D-penicillamine initiated
Pre-LT (March 2025)	Decompensated cirrhosis, GI bleeding, refractory ascites, severe cachexia (BMI 14.9), Child–Pugh C, MELD-Na 23	Loss of speech, severe dysarthria, bedridden, fixed contractures, dysphagia, sialorrhea, encephalopathy; UWDRS 162	End-stage liver disease	Nutritional support, repeated paracentesis, transplant evaluation
LT (April 3, 2025)	Living donor right lobe liver transplantation (uncle donor), GRWR 1.5%	Severe neurological disability	Successful graft implantation	LDLT
POD 4–10	Aspiration pneumonia requiring re-intubation and mechanical ventilation	No major neurological improvement yet	Improving graft function	Antibiotics, respiratory support, physical rehabilitation
POD 22	Discharged from hospital	Mild early neurological improvement	Stable graft function	Continued rehabilitation
Two months after LT	Readmission	Wing-beating tremor, worsening dysarthria and dysphagia; MRI progression	ALT 63 U/L, AST 50 U/L, total bilirubin 9.6 μmol/L, INR 1.1, tacrolimus 7.0 ng/mL; no evidence of graft dysfunction	D-penicillamine restarted
Eight months after LT	Stable outpatient follow-up	Progressive neurological improvement	Stable graft function	D-penicillamine discontinued after 6 months of therapy
Twelve months after LT	Returned to school and social activities	Fluent speech, ambulates with support, reduced contractures, independent feeding; MRI regression; UWDRS 59	Normal graft function	Ongoing rehabilitation and immunosuppression

LT, liver transplantation; LDLT, living donor liver transplantation; GRWR, graft-to-recipient weight ratio; POD, postoperative day; UWDRS, Unified Wilson Disease Rating Scale.

## Discussion

Wilson disease with a predominant neurological phenotype continues to represent one of the most challenging variants of the disorder, particularly in patients with rapidly progressive basal ganglia injury, severe movement impairment, and psychiatric and cognitive decline. Contemporary guidelines from the EASL-ERN consortium emphasize that liver transplantation (LT) remains an established therapy for acute liver failure and hepatic decompensation in Wilson disease, while its role in severe neurological involvement is still debated due to concerns regarding the irreversibility of central nervous system injury and the unpredictable neurological trajectory following restoration of copper homeostasis (3).

Despite these reservations, accumulating evidence suggests that meaningful neurological recovery following LT is possible. The comprehensive systematic review by Litwin et al., which included 48 studies encompassing 302 neurologically symptomatic patients, demonstrated substantial neurological improvement in 71.2% of cases and complete stability in an additional 6.9% (5). Only 7.9% experienced worsening, most often attributed to calcineurin-inhibitor neurotoxicity or uncontrolled preoperative damage. Importantly, the authors noted a clear trend toward more favorable outcomes in recent decades, likely reflecting improved perioperative management, more cautious immunosuppressive regimens, and refined patient selection.

Additional evidence is provided by Aksoy et al. (4), who reported resolution or marked improvement of neurological dysfunction after LT in 24 patients with Wilson disease and cirrhosis, as quantified using the Medici Scoring System. All surviving patients demonstrated clinically meaningful functional gains, with enhanced independence in activities of daily living within the first year after transplantation (7). These findings contribute to the growing literature supporting the reversibility of neurological deficits once toxic copper exposure is halted.

Several publications describe improvement even in extremely severe forms of the disease. Thus, in a series by Poujois et al., patients with severe neurological dysfunction (median UWDRS 96) demonstrated significant improvement after LT; the median UWDRS decreased from 96 to 38, and improvement in brain MRI was noted in 75% of patients (decrease in basal ganglia damage scores) (8). These data are consistent with our observations, including changes in MRI and significant functional improvement.

Neurological worsening observed 2 months after transplantation is consistent with the delayed deterioration reported in neuro-Wilson disease despite adequate graft function (2). In our patient, liver biochemistry and coagulation parameters remained stable, and tacrolimus trough levels were within the therapeutic range at the time of deterioration, making graft dysfunction and immunosuppressant-related neurotoxicity less likely explanations. The favorable response to reintroduction of D-penicillamine supports the hypothesis of transient copper redistribution or delayed copper-related neurotoxicity.

The marked neurological improvement in our patient is consistent with reports indicating that severe basal ganglia injury may retain partial reversibility once systemic copper overload is corrected. Several factors may account for the favorable outcome observed in this case. First, the patient underwent transplantation during an active and rapidly progressive phase before complete structural destruction of basal ganglia pathways occurred. Although the available evidence does not provide formal statistical confirmation, several case series summarized by Litwin et al. suggest that younger patients and those transplanted early after rapid neurological decline may experience more favorable outcomes. This likely reflects the partially reversible metabolic nature of copper-induced neuronal dysfunction in the early phases of the disease (5).

Second, the perioperative management avoided one of the key causes of post-transplant neurological worsening reported in the literature—calcineurin-inhibitor neurotoxicity (9, 10). In our case tacrolimus was deliberately initiated at minimal doses, with careful monitoring. Similar strategies are highlighted in Litwin et al. as crucial in preventing *de novo* neurological deterioration after LT.

Third, severe malnutrition and sarcopenia, which are known predictors of poor recovery in neurological Wilson disease, were aggressively treated pre- and postoperatively with albumin correction, high-calorie feeding, and structured nutritional support. Nutritional rehabilitation is essential for maximizing neuroplastic potential after LT (11, 12).

Finally, the patient underwent uninterrupted neuro–psycho–social rehabilitation starting in the ICU and continuing throughout hospitalization and follow-up. Literature consistently emphasizes that neurological improvement after LT is not solely a biological consequence of copper normalization but depends critically on intensive post-transplant rehabilitation. Cases described in multiple reports similarly document that the greatest functional gains occur in patients who receive structured physiotherapy and cognitive rehabilitation (4, 5, 13, 14).

Taken together, contemporary guidelines, retrospective series, and the largest available systematic review all indicate that LT can result in significant neurological recovery in selected patients with severe neuro-Wilson disease (15). Although transplantation remains controversial in the presence of profound neurological disability, emerging evidence supports individualized decision-making, particularly when deterioration is rapid, the patient is young, and irreversible structural brain damage has not yet occurred. The successful neurological and functional recovery documented in our case reinforces this evolving perspective and contributes additional evidence supporting the reversibility of severe neurological impairment following timely liver transplantation.

## Conclusion

This case illustrates that significant neurological recovery is achievable even in severely disabled adolescents with advanced neuro-Wilson disease. Restoration of copper metabolism through liver transplantation, combined with careful perioperative management and structured rehabilitation, can lead to substantial improvements in motor, cognitive, and functional status. Our findings support the view that severe neurological impairment should not be considered an absolute contraindication to transplantation in carefully selected patients.

## Data Availability

The original contributions presented in the study are included in the article/Supplementary Material, further inquiries can be directed to the corresponding author.
